# The complete chloroplast genome of *Menispermum dauricum* (Menispermaceae, Ranunculales)

**DOI:** 10.1080/23802359.2018.1501306

**Published:** 2018-08-17

**Authors:** Faiza Hina, Zhenyu Jin, Zhaoping Yang, Pan Li, Chengxin Fu

**Affiliations:** a Key Laboratory of Conservation Biology for Endangered Wildlife of the Ministry of Education, and Laboratory of Systematic and Evolutionary Botany and Biodiversity, College of Life Sciences, Zhejiang University, Hangzhou, China;; b College of Life Sciences, Tarim University, Alaer, China

**Keywords:** *Menispermum dauricum*, Menispermaceae, Papaveraceae, Ranunculales

## Abstract

*Menispermum dauricum* is a woody liana with great medicinal value. In the current study, we assembled the first chloroplast (cp) genome of *M. dauricum.* The whole chloroplast genome is 158,623 bp in length, with one large copy region (LSC: 88,879 bp), a small single copy region (SSC: 20,644 bp), and two inverted repeats (IR: 24,550 bp). The cp genome contains 114 unique genes with 80 protein-coding genes, 30 tRNA genes, and four rRNA genes. In our phylogeny of Ranunculales, Papaveraceae is found to be the basal group of Ranunculales and *M. dauricum* is sister to *Stephania japonica*.

The Asian moonseed (*Menispermum dauricum* DC.) is a woody liana widely distributed in East Asia from Central China to Siberia, Korean Peninsula, and Japan. Its rhizomes, which are known as ‘Bei-Dou-Gen’, have long been used in traditional Chinese medicine (TCM) as an analgesic and antipyretic, to treat colitis, dysentery, and rheumatic arthralgia, and also cardiovascular and thrombosis disorders (Zhao et al. 2012). Until now, genomic data for this important medicinal plant are scarce. Here, we sequenced the complete chloroplast genome of *M. dauricum* and analysed its phylogenetic position in Ranunculales.

Fresh leaf materials of *M. dauricum* were collected from China, Hebei Province, Qinghuangdao City, Zushan Town, Shanshenmiao Village, Beigou. The voucher specimen (*Pan Li LP161437*) was deposited in the Zhejiang University Herbarium (HZU). DNA was extracted from the silica gel dried leaves, using DNA Plantzol Reagent (Invitrogen, Shanghai, China) according to the manufacturer’s protocol. Whole chloroplast genome constructed with de novo assembly and mapped to the reference genome *Stephania japonica* (KU204903; Sun et al. 2016) using CLC v 10.1.1 (QIAGEN Bioinformatics, Redwood City, CA, United States). The whole sequence was annotated with Geneious 11.0.2 (Biomatters Ltd., Auckland, New Zealand) and then submitted to GenBank (MH298220).

The whole chloroplast genome of *M. dauricum* is 158,623 bp in length, with one large copy region (LSC: 88,879 bp), a small single copy region (SSC: 20,644 bp), and two inverted repeats (IR: 24,550 bp). The overall GC content of the genome is 38%. The cp genome consists of total 114 genes with 80 protein-coding genes, 30 tRNA genes, and four rRNA genes. There are 20 genes (eight tRNA genes and 12 protein-coding genes) with one intron and three genes (*rps12*, *clpP*, and *ycf3*) with two introns. *Ycf1* and *rps19* are the pseudogenes crossing the SSC/IR and LSC/IR borders. The *rps12* gene is trans-spliced, with the 5′ end located in the LSC and the 3′ end duplicated in the IR regions.

The phylogenetic tree were constructed using both maximum likelihood (ML) and Bayesian inference (BI) methods, with *Sabia yunnanensis* (KU204902; Sun et al. 2016) as the outgroup. Maximum likelihood analysis was carried out using RAxML-HPC2 on XSEDE v8.2.10 [AQ1] (Stamatakis 2014). Bayesian inference analysis was run with MrBayes on XSEDE v3.2.6 (Ronquist and Huelsenbeck 2003). Both ML and BI analyses were implemented on the CIPRES Science Gateway V. 3.3 (Miller et al. 2010) and generated the same tree topology (Figure 1). In the phylogeny of Ranunculales, Papaveraceae is revealed as the basal group, while *M. dauricum* is sister to *S. japonica*.

**Figure 1.**
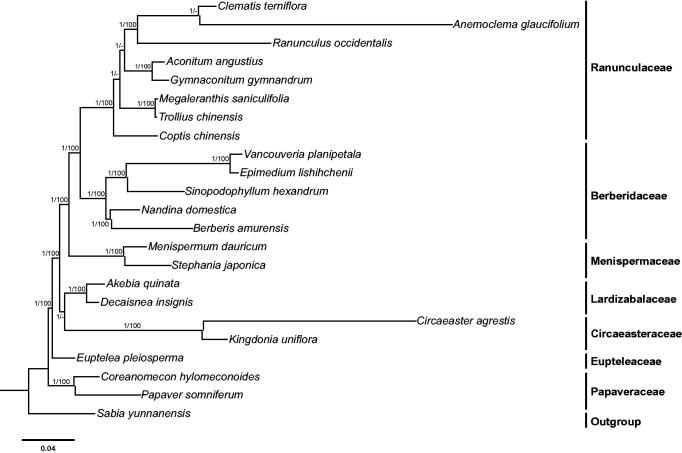
Molecular phylogeny of Ranunculales based on 23 complete cp genomes. The accession numbers are listed as follows: *Aconitum angustius* (MF155664); *Akebia quinata* (KX611091); *Anemoclema glaucifolium* (MG010811); *Berberis amurensis* (KM057374); *Circaeaster agrestis* (KY908400); *Clematis terniflora* (KJ956785); *Coptis chinensis* (KY120323); *Coreanomecon hylomeconoides* (KT274030); *Decaisnea insignis* (KY200671); *Epimedium lishihchenii* (KU522472); *Euptelea pleiosperma* (KU204900); *Gymnaconitum gymnandrum* (KT964697); *Kingdonia uniflora* (KY908401); *Megaleranthis saniculifolia* (FJ597983); *Menispermum dauricum* (MH298220); *Nandina domestica* (DQ923117); *Papaver somniferum* (KU204905); *Ranunculus occidentalis* (KX557270); *Sinopodophyllum hexandrum* (KR779994); *Stephania japonica* (KU204903); *Trollius chinensis* (KX752098); *Vancouveria planipetala* (MH337373); *Sabia yunnanensis* (KU204902).

In conclusion, this is the first report of the complete cp genome of *M. dauricum*, and will be useful for future phylogenomic studies.
